# Case of tuberculous pericarditis presenting as a giant pericardial adhesion and masquerading as a pericardial tumour

**DOI:** 10.4102/sajr.v22i1.1359

**Published:** 2018-09-27

**Authors:** Margaret E. Kisansa, Nndweleni M. Bida, Pule Mutati, Peter S. Ramoroko

**Affiliations:** 1 Department of Radiology, School of Medicine, Sefako Makgatho Health Sciences University, Dr George Mukhari Hospital, South Africa; 2 Department of Pathology, School of Medicine, Sefako Makgatho Health Sciences University, Dr George Mukhari Hospital, South Africa; 3 Department of Medicine, School of Medicine, Sefako Makgatho Health Sciences University, Dr George Mukhari Hospital, South Africa; 4 Department of Cardiothoracics, School of Medicine, Sefako Makgatho Health Sciences University, Dr George Mukhari Hospital, South Africa

## Abstract

Chronic pericarditis characterised by adhesions between the parietal and visceral pericardium is called adhesive pericarditis. In South Africa, tuberculosis is the most common cause of chronic pericarditis. We report a case of adhesive pericarditis that mimicked a tumour.

## Introduction

Tuberculous pericarditis is a common disease among the African and Asian populations because of the high prevalence of tuberculous infections.^1^ Chronic pericarditis characterised by adhesion between the parietal and visceral pericardium is called adhesive pericarditis. We report a case of a young female patient who presented with adhesive pericarditis that mimicked a pericardial tumour.

## Clinical history

A 27-year-old female patient presented with swelling of the abdomen and difficulty in breathing. She had a history of a tuberculous pericardial effusion one year prior to admission and had completed a regimen consisting of six months of anti-tuberculous therapy and steroids.

Clinical examination revealed a patient in respiratory distress, with congested neck veins and massive ascites. She had mild pallor, tachycardia and pedal oedema. The lungs were clear. The full blood count showed a low haemoglobin 9.7 g/dL, a low red cell count 3.30 × 10^12^/L and a normal white cell count. The renal function tests were grossly normal, and this patient did not have the human immunodeficiency virus (HIV). Electrocardiogram (ECG) showed right axis deviation with inverted T waves in leads II, III, aVF, V1–V6. The patient had features of constrictive pericarditis.

The echocardiography report was unresolved and confusing with a conclusion of a suspected tumour or thrombus in the right ventricle. Cardiac catheterisation study showed that the right ventricular (RV) and left ventricular (LV) pressures were consistent with constrictive pericarditis. The patient was then referred for magnetic resonance imaging (MRI) to further evaluate for the suspected tumour and ventricular function.

## Magnetic resonance imaging findings

**FIGURE 1:**
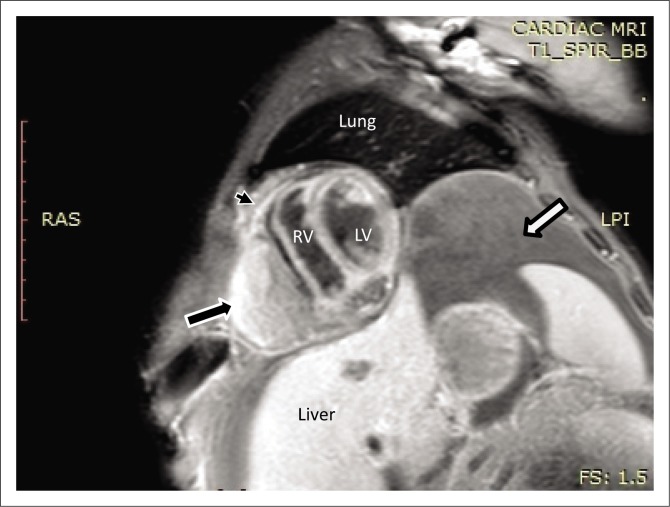
Short axis post contrast T1 spectral presaturation inversion recovery black blood sequence showing a high signal mass lesion (black arrow) with an associated nodular pericardium (black arrowhead) and massive ascites (white arrow).

**FIGURE 2:**
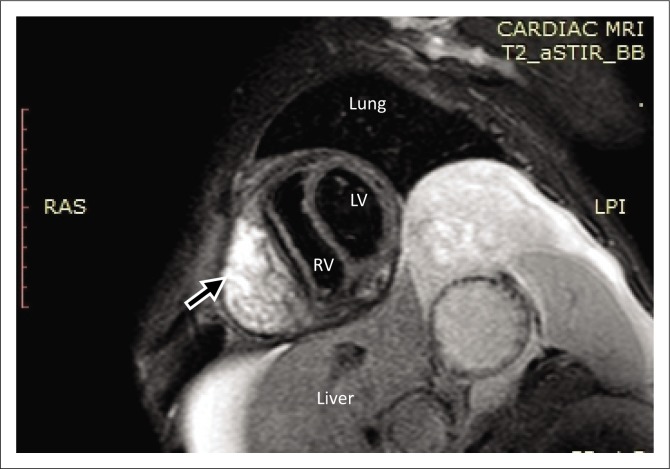
The lesion (black arrow) remains hyperintense on the T2 weighted short axis series.

**FIGURE 3:**
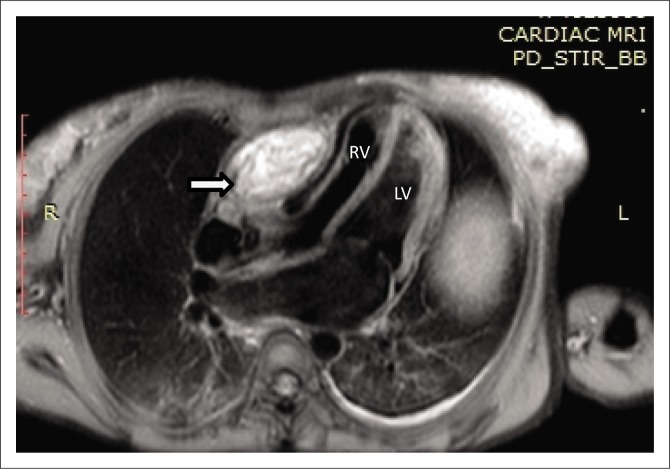
Proton density (4-chamber view) shows a predominantly hyperintense lesion with mass effect, as indicated by the white arrow.

Magnetic resonance imaging was performed on a Philips Multiva 1.5 Tesla scanner. Images were acquired in the para septal long axis, short axis and four-chamber views. Intravenous contrast was administered to further evaluate the mass lesion. The findings confirmed a large 6 cm × 4 cm × 8 cm mass compressing the right ventricle. The short axis post contrast T1 spectral presaturation inversion recovery black blood (T1_SPIR_BB) sequence image in Figure 1 showed a high signal intensity mass lesion compressing the right ventricle; this was associated with a nodular pericardium and massive ascites. The lesion was closely adherent to the pericardium but separable from the RV wall. The mass remained hyperintense on T2 short tau inversion recovery black blood (T2_STIR_BB) sequences (Figure 2). Proton density short tau inversion recovery black blood (PD STIR BB) series in Figure 3 also demonstrated a predominantly high signal intensity mass with hypointense streaks. The persistent high signal on both T1 and T2 sequences indicated the absence of fluid. Failure to suppress on all the fat suppression sequences, confirmed the absence of fat. There was marked mass effect, which impeded ventricular filling in diastole. A pronounced septal bounce was evident on the functional imaging series (not shown). The cardiac function was severely depressed with an ejection fraction (EF) of < 40%.

## Pathologic findings

**FIGURE 4:**
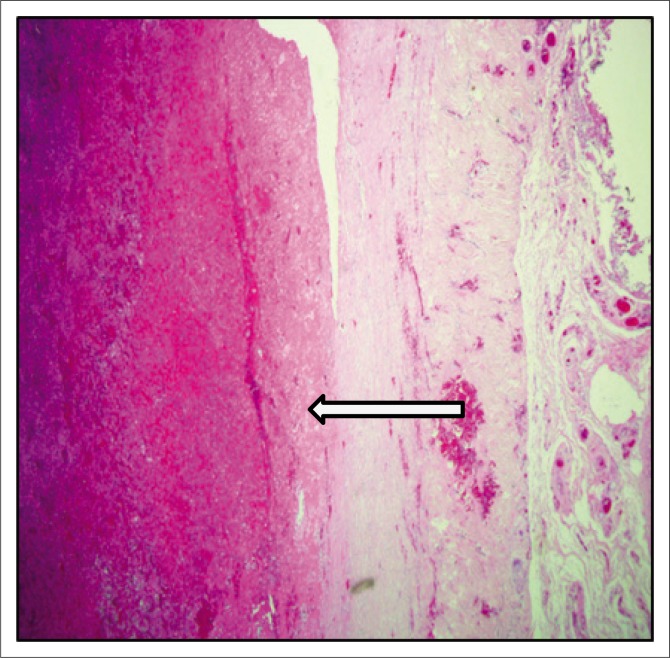
Haematoxylin and eosin (H&E) photomicrograph at 20× magnification of the fibrous pericardium showing organising fibrin deposition.

**FIGURE 5:**
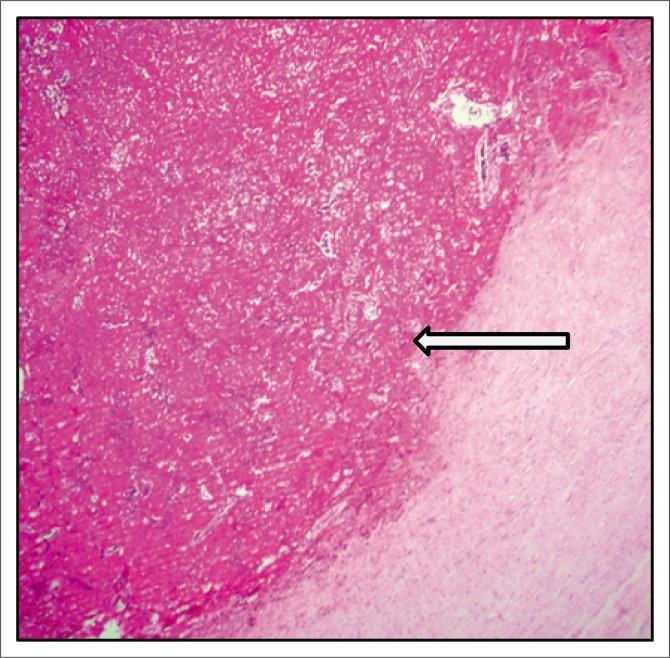
Haematoxylin and eosin (H&E) photomicrograph at higher magnification (40×) of the organising fibrin deposition showing an amorphous coagulum of acellular fibrin.

The patient subsequently underwent total pericardial stripping with excision of the mass. Histology showed fibrous pericardium with chronic inflammatory aggregates, including predominant lymphocytes with granulation tissue formation as seen in Figures 4 and 5. There was fibrinous exudate characterised by an eosinophilic meshwork of threads and amorphous coagulum, without a granulomatous response.

## Discussion

Pericarditis is defined as inflammation of the pericardium, and this has a number of causative agents like infection, neoplastic conditions, systemic conditions including collagen vascular diseases, trauma and others.^1^ Morphologically, the manifestations of pericardial disease include fibrin, fluid (serous, blood and lymph), purulent matter, fibrous tissue, neoplasm, granuloma, calcium and cholesterol.^2^ These conditions present with different clinical and radiological manifestations, as well as a diversity of causal agents as indicated above. Cherian (2004) reported that it was common to find pericardial effusions of unknown aetiology with an incidence of approximately 11% – 32% in countries like France, the United Kingdom (UK) and the United States (US).^1^ In South Africa, however, tuberculous pericarditis is the commonest cause of pericardial inflammation and effusions, with an incidence of 69.5%.^3^

Tuberculosis is a multisystem disease, which is uncommon in developed countries, with an incidence reported at 4%.^3^ On the contrary, its prevalence in developing countries is very high, and HIV has led to a further increase in the numbers of affected individuals.^1,4^

Tuberculosis pericarditis, caused by *Mycobacterium tuberculosis*, results from infection of the pericardium, most commonly by haematogenous or retrograde lymphatic spread.^1^ There are three clinico-pathological forms of chronic pericardial disease, viz. adhesive pericarditis, adhesive mediastino-pericarditis and constrictive pericarditis.^5^ Adhesive pericarditis is characterised by adhesion between the parietal and visceral pericardium.^5,6^ The disease is seen commonly in low socio-economic settings as a result of rheumatic heart disease, tuberculosis or, less commonly, pyogenic infections.^7^ In a small proportion of cases, the cause is unknown.^7^ The second form of chronic pericardial disease may occur together with mediastinal involvement, referred to as adhesive mediastino-pericarditis.^5^ In this instance, the pericardial sac is obliterated because of adhesion between the two layers of the pericardium, as well as between the parietal pericardium and surrounding mediastinal structures, chest wall and diaphragm. The third form of chronic pericardial disease takes the form of constrictive pericarditis, characterised by marked thickening of the parietal pericardium, with less involvement of visceral pericardium causing constriction of the great vessels entering and leaving the heart.^5^ The pericardial space is obliterated by dense fibrous tissue, which is often calcified.^2,5^ In many instances, tuberculosis is the most common cause. Occasionally, pericardial heart disease may be because of pyogenic infection,^8^ and in some, the cause is unknown.^7^

In tuberculous pericarditis, granulomas may not be histologically apparent if the patient is treated. In this case, polymerase chain reaction (PCR) may be useful to establish the diagnosis.^1^ Tissue cultures, however, have been found to be the most reliable.^1,9^

Cardiac MRI demonstrates the morphologically thickened pericardium^10^ and demonstrates functional changes that occur secondary to constriction (constrictive pericarditis). These changes are because of systolic and diastolic dysfunction, which is a result of impaired ventricular filling.^7^

This patient presented with an unusual form of constrictive tuberculous pericarditis, which was due to a pericardial adhesion that presented as a compressive mass. This resulted in a focal mass-like adhesion causing compression of the right ventricle and RV dysfunction. Surgical treatment in the form of pericardiectomy was performed, and the postoperative period was uneventful. The diagnosis of pericarditis was confirmed at histology. The patient was clinically well at follow-up, 12 months after surgery.

## Conclusion

The diagnosis of pericardial adhesions should be included in the differential diagnosis of patients with pericardial effusions who suddenly present with a pericardial mass.
